# Acute aortic dissection presenting as crossed neurological symptoms: a case report

**DOI:** 10.3389/fmed.2026.1882843

**Published:** 2026-08-06

**Authors:** Shuai Yan, Xiaoxi Wu, Jinbei Yu

**Affiliations:** 1 Department of Neurological Functional Examination, Affiliated Hospital of Hebei University, Baoding, Hebei, China; 2 Department of Nutrition, Affiliated Hospital of Hebei University, Baoding, Hebei, China; 3 Department of Neurology, The First Affiliated Hospital of Zhengzhou University, Zhengzhou, Henan, China

**Keywords:** aortic dissection, crossed neurological symptoms, monoplegia, paralysis, peripheral polyneuropathy

## Abstract

Aortic dissection can present with a wide variety of symptoms depending on the transient arterial occlusion during propagation of the dissection. Neurological manifestation, including hemiplegia, paralysis, paraplegia, and monoplegia, have been reported previously. However, crossed neuropathy has never been found. In this case report, we present the case of a 14-year-old girl who presented with right upper limb numbness and weakness together with persistent left lower extremity weakness, which mimicked peripheral polyneuropathy. The patient was finally diagnosed with arterial dissection but unfortunately died. Aortic dissection can be easily misdiagnosed as Guillain–Barré syndrome (GBS); this case emphasizes the importance of identifying atypical neurological manifestations of aortic dissection. Early identification and timely treatment are crucial for improving survival rates and prognosis.

## Introduction

Aortic dissection is a life-threatening condition characterized by a tear in the aortic intima, allowing blood to enter the media and form a false lumen that spreads along the aortic wall. The clinical manifestations of aortic dissection vary greatly, with 17–40% of patients experiencing neurological complications and an increased mortality rate (1). These complications may include ischemic stroke, spinal cord ischemia, and peripheral neuropathy, depending on the affected aortic branch (2). Although hemiplegia, paraplegia, and monoplegia have been documented, cross-nerve symptoms involving ipsilateral upper limb and contralateral lower limb defects have not been reported in aortic dissection. Here, we describe a case to alert clinicians to this unusual manifestation and emphasize the diagnostic challenges it poses.

## Case report

A 14-year-old girl was admitted to the Neurology Department of The First Affiliated Hospital of Zhengzhou University (Zhengzhou, China) with a 1-day history of pain and weakness in the left lower extremity, as well as paroxysmal numbness in the right upper limb. She had experienced a cold 4 days prior to admission and had largely recovered by the time of hospitalization. Two days before presentation, she reported bilateral lower extremity discomfort after performing strenuous bent-knee sit-ups. Her medical history was significant for the surgical repair of a patent ductus arteriosus (PDA) at 3 months of age. There was no other relevant past medical history.

On admission, the patient was conscious but appeared unwell, with poor appetite and low energy. Vital signs were stable: temperature of 36.5 °C; pulse of 96 beats/min; respiratory rate of 15 beats/min; and blood pressure (BP) of 112/67 mmHg in the right arm (Table 1). The abdomen was soft, and the liver and spleen were not palpable below the costal margin. Cranial nerve examination was normal. The right upper limb was slightly cooler than the contralateral side, without cyanosis; the left lower extremity also felt cool to the touch. Motor examination revealed the following: the proximal strength of the right upper limb was V, while distal strength was V−; left upper limb and right lower limb strength were V; left lower limb proximal strength was IV+, and distal strength was IV. Deep tendon reflexes (biceps, triceps, knee, and ankle) were absent on the right upper limb and the left lower limb, but normal on the contralateral sides. Sensory examination revealed decreased pain, light touch, and temperature sensation below the left knee, with preserved vibration sense. Pathological reflexes were absent. The asymmetric, non-length-dependent pattern of weakness and areflexia—restricted to the affected limbs rather than generalized—was a critical clue that pointed away from typical polyneuropathy.

**Table 1:** Patient’s vital signs on admission.

Parameter	Value	Reference range
Vital signs		
Temperature	36.5 °C	36.0–37.2 °C
Heart rate	96 beats/min	60–100 beats/min
Respiratory rate	15 breaths/min	12–20 breaths/min
Blood pressure (right arm)	112/67 mmHg	—
Blood pressure (left arm)	124/72 mmHg	—

In addition to the neurological examination, a thorough vascular assessment was performed. Capillary refill time was slightly prolonged in the right hand (3 s) and the left foot (3 s) compared to the contralateral sides (2 s). No bruits were auscultated over the carotid, abdominal, or femoral arteries. While these findings were not overtly specific, they raised the possibility of a vascular etiology.

Notable laboratory findings on admission included D-dimer of 1.47 mg/L (reference: <0.5 mg/L), fibrin degradation products (FDPs) of 14.68 mg/L (reference: <5.0 mg/L), white blood cell count of 15.87 × 10^9^/L (reference: 4.0–10.0 × 10^9^/L), neutrophil percentage of 80.4% (reference: 40–75%), glutamic-oxaloacetic transaminase (GOT) of 78 U/L (reference: <40 U/L), creatine kinase (CK) of 1600.0 U/L (reference: 26–192 U/L), troponin I of 0.046 μg/L (reference: <0.04 μg/L), and N-terminal pro-brain natriuretic peptide (NT-proBNP) of 218.90 pg./mL (reference: <125 pg./mL). The elevated CK and troponin I on admission suggested early myocardial and/or skeletal muscle ischemia, a finding that should have raised suspicion for a vascular event. Other laboratory values were within normal limits. Electrocardiogram (ECG) showed no abnormalities.

**Figure 1:**
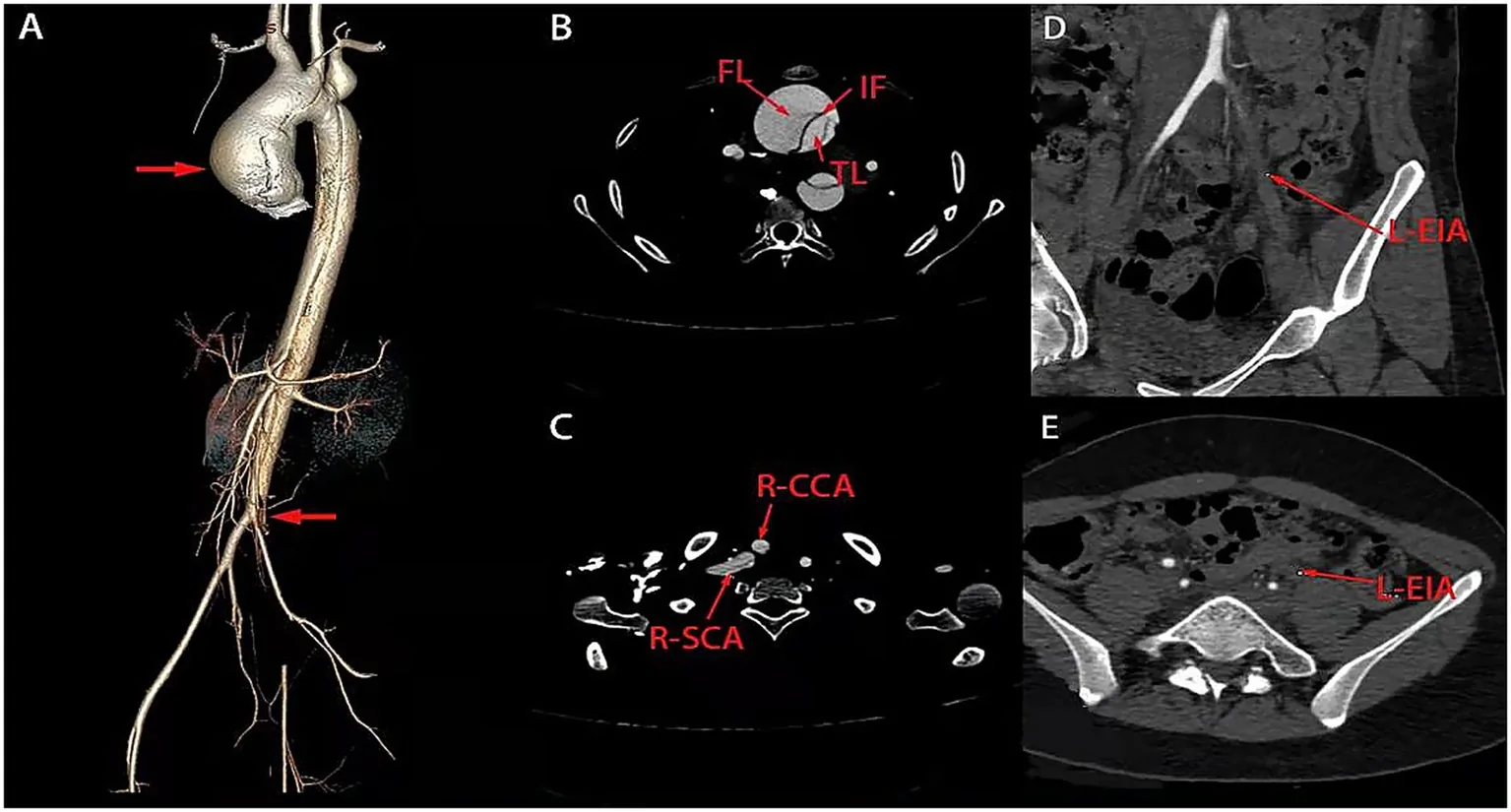
Computerized tomographic angiography (CTA) of the aorta artery (AA): **(A,B)** demonstrated that the ascending aorta enlarged markedly, with spiral tears throughout the endovascular range, from the root of AA to L-CLA; **(C)** two-chambered manifestation of R-SCA; **(D,E)** L-EIA was undeveloped in the CTA. L-CIA, left common iliac artery; L-EIA, left external iliac artery; R-CCA, right common carotid artery; R-SCA, right subclavian artery; TL, true lumen; FL, false lumen; IF, intimal flap.

Approximately 3 h after admission, the patient complained of precordial discomfort, chest tightness, and palpitations. Arterial blood gas analysis and repeat ECG findings were normal. However, color Doppler echocardiography revealed a floating membrane-like echo in the aortic arch and moderate pericardial effusion. Repeat cardiac enzyme measurements showed CK of 5,631 U/L, troponin I of 0.046 μg/L, and NT-proBNP of 305.94 pg./mL. Computed tomography angiography (CTA) demonstrated (Figure 1): (1) aortic dissection (Debakey type I) with marked dilation of the ascending aorta (Figures 1A,B); (2) occlusion of the proximal and middle segments of the left external iliac artery (L-EIA) (Figures 1D,E); (3) extension of the dissection of the right subclavian artery (R-SCA), with a double-lumen appearance with an intimal flap (Figure 1C); (4) patency of the right common carotid artery (R-CCA) without definite dissection; (5) mild inflammation in the right upper and left lower lung lobes; (6) bilateral pleural, pericardial, gallbladder fossa, and pelvic effusions; and (7) left kidney hypoperfusion.

The patient was immediately transferred to the Department of Cardiovascular Surgery and taken to the operating room. A drastic decrease in blood pressure following anesthesia suggested cardiac tamponade, which was confirmed upon thoracotomy. Marfan syndrome (MFS) was suspected intraoperatively due to the pronounced ascending aortic dilatation and the patient’s body habitus (tall stature and long fingers). However, no preoperative systemic assessment for MFS had been performed, and no genetic testing was undertaken. The patient had no known family history of MFS or other connective tissue disorders. Unfortunately, her blood pressure collapsed again postoperatively, and she died despite resuscitation efforts. An autopsy was not performed, which limited the ability to confirm the underlying etiology histologically.

## Discussion

In this report, we describe a 14-year-old girl who presented with a crossed pattern weakness of the right upper limb and left lower extremity, accompanied by sensory abnormalities in the affected limbs. The neurological findings—asymmetric, non-length-dependent, and with areflexia confined to the affected limbs—initially raised suspicion for peripheral neuropathy, such as Guillain–Barré syndrome (GBS) or other polyneuropathies (3). However, several features argue against GBS: the asymmetry of deficits, the restriction of areflexia to the affected limbs (with normal reflexes contralaterally), the presence of cool extremities, and the absence of the classical ascending pattern. The clinical picture was ultimately clarified by CTA, which revealed a DeBakey type I aortic dissection with involvement of the right subclavian artery and occlusion of the left external iliac artery. This case is notable for the crossed neurological presentation, which, to our knowledge, has not previously been reported in aortic dissection.

GBS is classically defined as a symmetrical, ascending acute polyradiculoneuropathy, often with diffuse areflexia. While atypical presentations—including asymmetry—have been described, particularly in chronic inflammatory demyelinating polyneuropathy (CIDP) (4), the constellation of findings in our patient was inconsistent with a primary demyelinating process. The absence of cerebrospinal fluid (CSF) analysis and nerve conduction studies/electromyography (NCS/EMG) in this case is acknowledged; however, the rapid clinical deterioration and the immediate need for surgical intervention preclude these investigations (5). We therefore emphasize that, in patients with asymmetric limb weakness, particularly when accompanied by vascular signs or a history of congenital heart disease, aortic dissection should be considered early in the differential diagnosis.

The mechanism of upper limb involvement in this case appears to have been dissection of the R-SCA, as demonstrated by the double-lumen appearance on CTA. This finding has been previously described as a cause of acute peripheral nerve ischemia, either through direct arterial occlusion or via compression by the false lumen (6). The right upper limb symptoms—intermittent numbness and mild distal weakness—are consistent with early or intermittent ischemia, as sensory fibers are typically more sensitive to hypoperfusion than motor fibers (7). The left lower extremity weakness, on the other hand, resulted from occlusion of the L-EIA, as clearly demonstrated on CTA. The bilateral lower extremity pain following exercise may have represented early ischemic symptoms, although the history of strenuous activity initially distracted attention from this possibility (8, 9).

The epidemiology of aortic dissection in children and adolescents differs markedly from that of adults. While hypertension is the predominant risk factor in older populations, pediatric aortic dissection is rare and is most commonly associated with congenital heart disease (such as coarctation of the aorta and PDA), connective tissue disorders (including Marfan syndrome, Loeys–Dietz syndrome, and Ehlers–Danlos syndrome), and trauma (10, 11). In our patient, prior PDA repair represented a significant risk factor, although the exact contribution of this lesion to the development of aortic dissection is uncertain. The intraoperative suspicion of MFS was based on aortic morphology and body habitus; however, the absence of genetic testing and family history left the etiology unconfirmed. The lack of an autopsy further limited our ability to determine the underlying pathology. We acknowledge this as a major limitation of the report.

The diagnosis of aortic dissection in this case was delayed because of the atypical neurological presentation. Patients with painless or neurologically predominant aortic dissection are at high risk of misdiagnosis (12). The presence of elevated D-dimer and CK, along with troponin elevation, should have prompted early vascular imaging. In retrospect, the combination of asymmetric limb weakness, vascular signs, elevated CK (suggesting muscle ischemia), and a history of congenital heart disease should have raised a higher index of suspicion for aortic dissection (13).

This case also underscores the importance of a thorough vascular examination in patients presenting with asymmetric neurological deficits. In the acute setting, bedside vascular assessment may be the only available tool before advanced imaging can be obtained (14).

The clinical presentation of aortic dissection can mimic a wide range of neurological conditions, including GBS, acute ischemic stroke, spinal cord infarction, and peripheral nerve entrapment. The crossed pattern observed in this case—involving the right upper and left lower limbs—is particularly deceptive, as it does not conform to any single spinal cord, brainstem, or peripheral nerve territory (15). This pattern should prompt consideration of a multifocal vascular or ischemic process rather than a primary neurological disease (16).

## Limitations

This case report has several limitations. First, no CSF analysis or NCS/EMG was performed as the patient’s rapid deterioration and need for urgent surgery precluded these studies. Second, genetic testing for connective tissue disorders was not obtained, and the family history was incomplete. Third, an autopsy was not performed, preventing histological confirmation of aortic wall pathology and definitive diagnosis of MFS. Fourth, the absence of Doppler ultrasound or dedicated vascular imaging of the right upper limb limited the characterization of the subclavian artery involvement. Finally, the manuscript was not originally structured according to the CAse REport (CARE) guidelines; we have revised it to comply with these standards.

## Conclusion

Peripheral nerve involvement in aortic dissection is relatively rare, and crossed neurological manifestations have not been previously reported. This case demonstrates that aortic dissection can present with misleading neurological symptoms that mimic GBS or other polyneuropathies, particularly in young patients. A high index of suspicion, careful vascular examination, and prompt imaging are essential for early diagnosis. Given the high mortality of aortic dissection, timely recognition and surgical intervention are critical to improve outcomes.

## Data Availability

The raw data supporting the conclusions of this article will be made available by the authors, without undue reservation.
